# Tetracarbonatodiruthenium Fragments and Lanthanide(III) Ions as Building Blocks to Construct 2D Coordination Polymers

**DOI:** 10.3390/polym11030426

**Published:** 2019-03-05

**Authors:** Daniel Gutiérrez-Martín, Miguel Cortijo, Álvaro Martín-Humanes, Rodrigo González-Prieto, Patricia Delgado-Martínez, Santiago Herrero, José L. Priego, Reyes Jiménez-Aparicio

**Affiliations:** 1 Departamento de Química Inorgánica, Facultad de Ciencias Químicas, Universidad Complutense de Madrid, Ciudad Universitaria, E-28040 Madrid, Spain; dangut04@ucm.es (D.G.-M.); miguelcortijomontes@ucm.es (M.C.); alvama09@ucm.es (Á.M.-H.); rodgonza@ucm.es (R.G.-P.); 2 Centro de Asistencia a la Investigación Difracción de Rayos X, Facultad de Ciencias Químicas, Universidad Complutense de Madrid, E-28040 Madrid, Spain; patriciadelgado@ucm.es (P.D.-M.)

**Keywords:** diruthenium compounds, lanthanide complexes, coordination polymers, magnetic properties

## Abstract

Two-dimensional coordination polymers of [Pr(DMSO)_2_(OH_2_)_3_][Ru_2_(CO_3_)_4_(DMSO)(OH_2_)]·5H_2_O (**Prα**) and [Ln(OH_2_)_5_][Ru_2_(CO_3_)_4_(DMSO)]·*x*H_2_O (Ln = Sm (**Smβ**), Gd (**Gdβ**)) formulae have been obtained by reaction of the corresponding Ln(NO_3_)_3_·6H_2_O dissolved in dimethyl sulphoxide (DMSO) and K_3_[Ru_2_(CO_3_)_4_]·4H_2_O dissolved in water. Some DMSO molecules are coordinated to the metal atoms reducing the possibilities of connection between the [Ru_2_(CO_3_)_4_]^3−^ and Ln^3+^ building blocks giving rise to the formation of two-dimensional networks. The size of the Ln^3+^ ion and the synthetic method seem to have an important influence in the type of two-dimensional structure obtained. Slow diffusion of the reagents gives rise to **Prα** that forms a 2D net that is built by Ln^3+^ ions as triconnected nodes and two types of Ru_2_^5+^ units as bi- and tetraconnected nodes with (2-c)(3-c)_2_(4-c) stoichiometry (α structure). An analogous synthetic procedure gives **Smβ** and **Gdβ** that display a grid-like structure, (2-c)_2_(4-c)_2_, formed by biconnected Ln^3+^ ions and two types of tetraconnected Ru_2_^5+^ fragments (β structure). The magnetic properties of these compounds are basically explained as the sum of the individual contributions of diruthenium and lanthanide species, although canted ferrimagnetism or weak ferromagnetism are observed at low temperature.

## 1. Introduction

The first tetracarbonatodiruthenium compound, {Na_3_[Ru_2_(CO_3_)_4_]·6H_2_O}_n_, was described by Wilkinson et al. several years ago [1] although their crystal structure was reexamined by Cotton et al. [2] in order to elucidate the relationship between diruthenium(II,III) and diruthenium(III,III) carbonato complexes.

**Figure 1:**
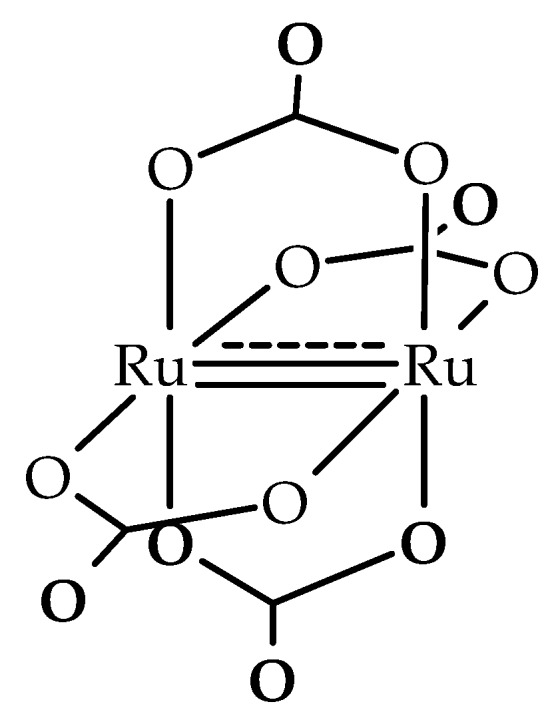
Representation of the [Ru_2_(CO_3_)_4_]^3−^ anion.

The very stable carbonate anion, [Ru_2_(CO_3_)_4_]^3−^, has two ruthenium atoms joined by four bridging carbonate ligands forming the typical paddlewheel structure with a Ru–Ru bond order of 2.5 (Figure 1). This anion is very similar to the diruthenium [Ru_2_(O_2_CR)_4_]^+^ cation and, in accordance with the theoretical calculations carried out by Norman et al. [3], a configuration σ^2^π^4^δ^2^π*^2^δ*^1^ is assumed. Due to the near degeneracy of the π* and δ* orbitals, the diruthenium(II,III) complexes with paddlewheel structure usually present three unpaired electrons (S = 3/2) [3,4,5,6]. A magnetic study shows that this complex presents a canted ferrimagnet behavior below 4.2 K [7].

As can be observed in Figure 1, in the [Ru_2_(μ-O_2_CO)_4_]^3−^ anion each carbonate ligand has one free oxygen atom. This oxygen atom can be coordinated to other metal atoms giving heterometallic compounds. Thus, the combination of diruthenium dimers with S = 3/2 and metal complexes with different spins leads to solids with interesting magnetic properties [8,9,10,11,12,13,14,15,16,17,18]. Thus, the reaction of K_3_[Ru_2_(CO_3_)_4_]·4H_2_O and Ni(NO_3_)_2_·6H_2_O forms a 3D network with magnetic order at very low temperatures [8]. Three-dimensional networks of H_x_K_1−x_M^II^[Ru_2_(CO_3_)_4_](H_2_O)_y_(MeOH)_z_(M = Mn, Fe, Co, Ni, Mg) stoichiometry were obtained by the reaction of K_3_[Ru_2_(CO_3_)_4_]·4H_2_O with M^2+^ salts (M = Mn, Co, Ni, Cu, Fe, Mg). These compounds show magnetic order as canted ferrimagnets with very similar ordering temperatures but it has been proposed that the presence of M(II) cations does not significantly contribute to the magnetic coupling pathways [9].

The influence of chloride and bromide anions on the self-assembling of [Ru_2_(CO_3_)_4_]^3−^ and Co^2+^ or Cu^2+^ ions in aqueous solution has been studied. This self-assembling leads to layer structures with different composition: [{Co(H_2_O)_4_}_2_Ru_2_(CO_3_)_4_(H_2_O)Cl]_n_·7.5nH_2_O, [{Co(H_2_O)_4_}_2_Ru_2_(CO_3_)_4_(H_2_O)_2_]_n_·[{Co(H_2_O)_4_}_2_Ru_2_(CO_3_)_4_Br_2_]_n_·10.5nH_2_O [10] and K_2_Li[Cu(H_2_O)_2_Ru_2_(CO_3_)_4_X_2_]·5H_2_O [X = Cl, Br) [11].

Other heterometallic complexes with metals as Co^2+^ [12], Cd^2+^ [13], Zn^2+^ [14] and Mn^2+^ [14,15,16,17] have been described displaying a great versatility to form different networks. Interestingly, the complex Mn_4_(H_2_O)_16_H[Ru_2_(CO_3_)_4_]_2_[Ru_2_(CO_3_)_4_(H_2_O)_2_]·11H_2_O [18] is a soft ferromagnet (*T*_c_ = 3 K) and K[Mg(H_2_O)_4_Ru_2_(CO_3_)_4_]·H_2_O shows magnetic ordering below 3.5 K and its coercivity improves when the particle size changes from the micrometer to the nanometer scale [19].

However, the number of complexes containing tetracarbonatodiruthenium and lanthanides species are very scarce. The first heteronuclear complexes of the type Ln[Ru_2_(CO_3_)_4_]·8H_2_O (Ln = Gd, Nd, Ho, Yb) were described by Miller et al. [20] although only microcrystalline solids were isolated. However, very recently the formation of single crystals of the complexes [Ln(OH_2_)_4_][Ru_2_(CO_3_)_4_(OH_2_)]·xH_2_O [Ln = Gd, Eu, Yb] and K_3_[Gd(H_2_O)_4_]_2_[Ru_2_(CO_3_)_4_]_3_·3.5H_2_O has been achieved. The resolution of the crystal structures of these compounds shows in all cases the formation of 3D coordination polymers [21].

In order to block some coordination positions and to obtain polymers with lower dimensionality than the previous 3D-compounds, we used dimethyl sulphoxide, which is a solvent with a strong donor character. Thus, using this solvent we prepared two-dimensional coordination polymers of the type [Pr(DMSO)_2_(OH_2_)_3_][Ru_2_(CO_3_)_4_(DMSO)(OH_2_)]·5H_2_O (**Prα**) and [Ln(OH_2_)_5_][Ru_2_(CO_3_)_4_(DMSO)]·xH_2_O (Ln = Sm (**Smβ**), Gd (**Gdβ**)). Moreover, we also prepared [Ln(OH_2_)_4_][Ru_2_(CO_3_)_4_(OH_2_)]·xH_2_O (Ln = Pr (**Pr3D**), Sm (**Sm3D**)) for comparative reasons. These complexes display a 3D polymeric structure and they are isostructural to the Gd, Eu and Yb derivatives that were previously reported by our research group [21]. The magnetic properties and crystal structures of the new complexes are described in this paper.

## 2. Materials and Methods

### 2.1. Materials and Physical Measurements

K_3_[Ru_2_(CO_3_)_4_]·4H_2_O was prepared following a published procedure [2]. The rest of the reagents were purchased from commercial sources and used as received without further purification. Elemental analyses were done by the Microanalytical Services of the Universidad Complutense de Madrid. FTIR spectra were measured using a Perkin–Elmer Spectrum 100 with a universal ATR accessory in the 4000–650 cm*^−^*^1^ spectral range. Thermogravimetric measurements were perfomed using a PerkinElmer Pyris 1 TGA instrument under nitrogen atmosphere with a heating rate of 5 °C min^–1^. A Quantum Design MPMSXL Superconducting Quantum Interference Device (SQUID) magnetometer was used to obtain the variable temperature magnetic susceptibility data of finely ground crystals in the temperature range 2–300 K under 1 T. Magnetization measurements were collected at 2 K from −5 to 5 T. All data were corrected taking into account the signal of the sample holder and the diamagnetic contributions of the samples. The molar diamagnetic corrections were calculated on the basis of Pascal’s constants. Single crystal X-ray diffraction measurements were carried out with a Bruker Smart-CCD diffractometer at room temperature using a Mo Kα (λ = 0.71073 Å) radiation and a graphite monochromator. CCDC 1894711-1894714 contain the crystallographic data for the new compounds described in this work. These data can be obtained free of charge from the Cambridge Crystallographic Data Centre via www.ccdc.cam.ac.uk/data_request/cif. A summary of some crystal and refinement data are shown in Table 1. Powder X-ray diffraction (PXRD) measurements were carried out by the X-ray service of the UCM using a PANalytical X’Pert MPD diffractometer.

### 2.2. Synthesis

#### 2.2.1. Synthesis of [Pr(DMSO)_2_(OH_2_)_3_][Ru_2_(CO_3_)_4_(DMSO)(OH_2_)]·5H_2_O (**Prα**)

Brownish yellow crystals were obtained after a few days by slow diffusion of a 15 mL water solution of 0.095 mmol (0.06 g) of K_3_[Ru_2_(CO_3_)_4_]·4H_2_O into a solution of 0.11 mmol of the corresponding Ln(NO_3_)_3_·6H_2_O in 15 mL of DMSO. The water and DMSO solutions were separated by 10 mL of water. The crystals were filtered, washed with acetone (3 × 15 mL) and dried under vacuum: Yield: 0.04 g (43%). Anal. Calcd (%) for PrRu_2_C_10_H_26_O_19_S_3_·5H_2_O (979.62 g·mol^−1^): C, 12.26; H, 3.70. Found (%): C, 12.01; H, 3.53. FT-IR (cm^−1^): 1672sh, 1639w, 1504s, 1415sh, 1326m, 1314m, 1251m, 1058m, 1010m, 991sh, 965m, 935m, 813m, 715m.

#### 2.2.2. Synthesis of [Ln(OH_2_)_5_][Ru_2_(CO_3_)_4_(DMSO)]·3H_2_O (Ln = Sm (**Smβ**), Gd (**Gdβ**))

Orange crystals of these compounds were prepared following a similar procedure to that employed to prepare **Prα** using 0.11 mmol of the corresponding Ln(NO_3_)_3_·6H_2_O. (**Smβ)**: Yield: 0.07 g (90%). Anal. Calcd (%) for SmRu_2_C_6_H_16_O_18_S·3H_2_O (814.80 g·mol^−1^): C, 8.84; H, 2.72; S, 3.94. Found (%): C, 8.45; H, 2.74; S, 3.78. FT-IR (cm^−1^): 1659w, 1629w, 1498sh, 1449s, 1346m, 1291m, 1261m, 1054m, 985s, 920m, 812m, 708m. (**Gdβ**) Yield: 0.04 g (51%). Anal. Calcd (%) for GdRu_2_C_6_H_16_O_18_S·3H_2_O (821.68 g·mol^−1^): C, 8.77; H, 2.70; S, 3.90. Found (%): C, 8.48; H, 2.74; S, 3.68 FT-IR (cm^−1^): 1654w, 1626w, 1500sh, 1449s, 1347m, 1291m, 1261m, 1055m, 985m, 921m, 813m, 708m.

#### 2.2.3. Synthesis of [Ln(OH_2_)_4_][Ru_2_(CO_3_)_4_(OH_2_)]·xH_2_O (Ln = Pr (P**r3D**), Sm (**Sm3D**))

Method a: A solution of 0.32 mmol (0.20 g) of K_3_[Ru_2_(CO_3_)_4_]·4H_2_O in 20 mL of water was added dropwise to a solution of 0.35 mmol of the corresponding Ln(NO_3_)_3_·xH_2_O in 20 mL of water. The mixture was stirred overnight and the brown solid obtained was filtered, washed with water, methanol and diethyl ether and dried under vacuum. (**Pr3D**): Yield: 0.14 g (60%). Anal. Calcd (%) for PrRu_2_C_4_H_12_O_18_·2H_2_O (727.207 g·mol^−1^): C, 6.61; H, 2.22. Found (%): C, 6.24; H, 2.13. (**Sm3D**) Yield: 0.20 g (85%). Anal. Calcd (%) for SmRu_2_C_4_H_12_O_18_·2H_2_O (736.65 g·mol^−1^): C, 6.52; H, 2.19. Found (%): C, 6.20; H, 2.15. 

Method b: **Pr3D** and **Sm3D** were obtained after a few days by slow diffusion of a solution of 0.05 mmol (0.03 g) of K_3_[Ru_2_(CO_3_)_4_]·4H_2_O in 20 mL of water into a solution of 0.07 mmol of the corresponding Ln(NO_3_)_3_·xH_2_O in 15 mL of water. The two solutions were separated by 10 mL of water. The crystals were washed with water, methanol and diethyl ether. (**Pr3D**): Yield: 0.02 g (54%). Anal. Calcd (%) for PrRu_2_C_4_H_12_O_18_·3H_2_O (745.223 g·mol^−1^): C, 6.45; H, 2.43. Found (%): C, 6.14; H, 2.28. (**Sm3D**) Yield: 0.035 g (93%). Anal. Calcd (%) for SmRu_2_C_4_H_12_O_18_·3H_2_O (754.665 g·mol^−1^): C, 6.37; H, 2.40. Found (%): C, 6.15; H, 2.19. 

**Pr3D**: FT-IR (cm^−1^): 1651w, 1597w, 1494s, 1461s, 1320w, 1252m, 1051m, 814w, 762w, 716w. **Sm3D**: FT-IR (cm^−1^): 1649w, 1598w, 1503s, 1463s, 1255m, 1051w, 814w, 716w.

## 3. Results and Discussion

### 3.1. Synthesis

As previously reported, the equimolecular reaction of K_3_[Ru_2_(CO_3_)_4_]·4H_2_O and Ln(NO_3_)_3_·xH_2_O in water under different conditions (direct mixture, layering synthesis or solvothermal synthesis with or without microwave radiation) leads to the formation of 3D structures with [Ln(OH_2_)_4_][Ru_2_(CO_3_)_4_(OH_2_)]·xH_2_O composition [21]. This approach has been also successfully employed to prepare **Pr3D** and **Sm3D** in this work. In order to reduce the dimensionality of that structure, the same reaction was assayed by dissolving the lanthanide salts in a strong donor solvent such as dimethyl sulphoxide (DMSO) with the aim of blocking some coordination positions of the metals. The layering method was selected to prepare the new compounds because it was successfully used to obtain single crystals of [Ln(OH_2_)_4_][Ru_2_(CO_3_)_4_(OH_2_)]·xH_2_O. Changing the solvent of the rare earth salt solutions was sufficient to form two other structures, one with LnRu_2_(CO_3_)_4_·3DMSO·9H_2_O composition for the lighter lanthanide (Pr) and LnRu_2_(CO_3_)_4_·DMSO·8H_2_O composition for the heavier ones (Sm, Gd and Dy). The decrease of the lanthanide radius could be the explanation of that change.

**Figure 2:**
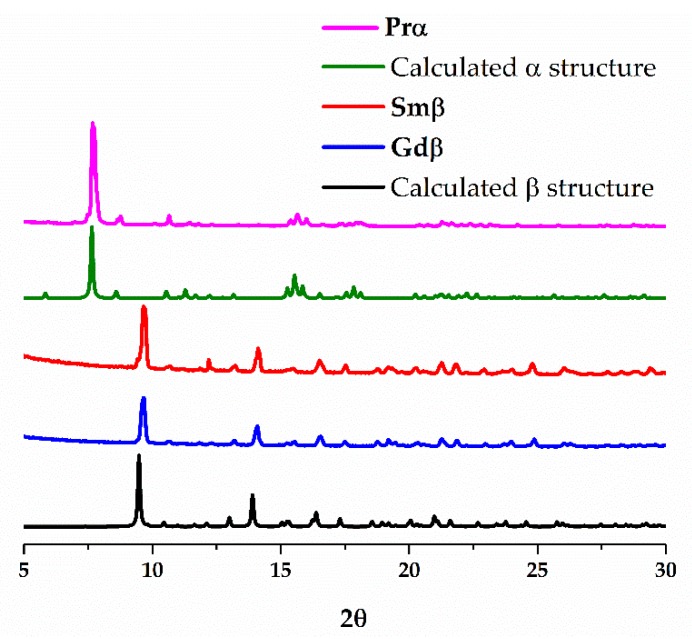
Calculated powder X-ray diffractograms for the 2D structures simulated from the single crystal data of **Prα** (α structure, green) and **Smβ** (β structure, black). Experimental powder X-ray diffraction pattern obtained for a bulk sample of **Prα** (pink), **Smβ** (red) and **Gdβ** (blue).

Before adding the water solution of K_3_[Ru_2_(CO_3_)_4_]·4H_2_O, 10 mL of neat water was added to avoid the precipitation of the compounds at the interface of both solutions. This procedure permits the slow diffusion of the reactants leading to the direct formation of single crystals, suitable for X-ray diffraction analysis, with acceptable yields. It should be also taken into account that the insolubility of the compounds prevents their recrystallization. Powder X-ray diffraction measurements show that a single phase is obtained; α structure in the case of **Prα** and β structure in the case of **Smβ** and **Gdβ** (Figure 2). The IR spectra of **Prα**, **Smβ** and **Gdβ** are shown in Figures S1 and S2.

**Figure 3:**
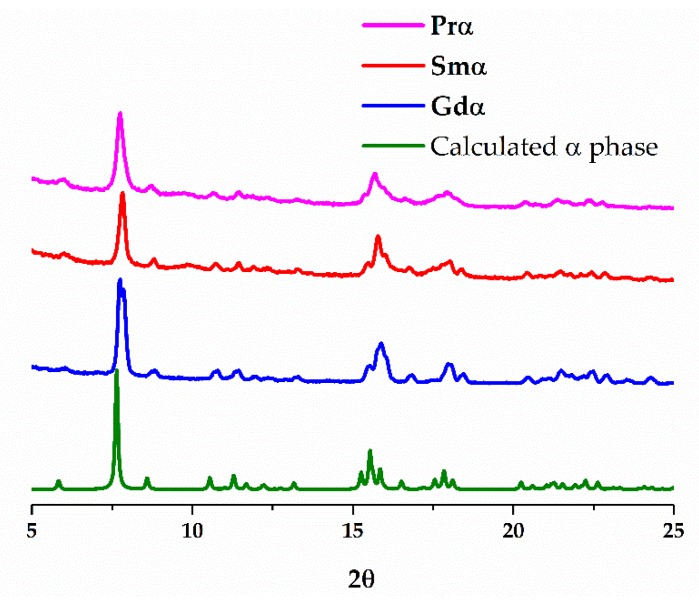
Theoretical powder X-ray diffractogram for the 2D α structure simulated from the single crystal data of **Prα** (green). Experimental powder X-ray diffraction pattern obtained for the bulk sample prepared by direct mixing of a 15 mL water solution of 0.095 mmol (0.06 g) of K_3_[Ru_2_(CO_3_)_4_]·4H_2_O into a solution of 0.11 mmol of the corresponding Ln(NO_3_)_3_·6H_2_O in 15 mL of DMSO (Pink: Ln = Pr. Red: Ln = Sm. Blue: Ln = Gd).

The direct mixing of the reagents, K_3_[Ru_2_(CO_3_)_4_]·4H_2_O in water and Ln(NO_3_)_3_·xH_2_O in DMSO, instantaneously produces precipitation of a solid that presents the same single phase for Pr, Sm and Gd, as demonstrated by powder X-ray diffraction analysis (Figure 3). This phase is the same as the one obtained for praseodymium by the layering method (α structure). However, no completely satisfactory elemental analyses have been obtained for these samples. Nevertheless, these results point out that LnRu_2_(CO_3_)_4_·3DMSO·9H_2_O is the kinetic compound, whereas LnRu_2_(CO_3_)_4_·DMSO·8H_2_O is thermodynamically more stable, as least when Ln = Sm and Gd.

### 3.2. Structural Description

The crystal structure of **Prα**, **Smβ** and **Gdβ** was determined from single crystal X-ray diffraction. They crystallize in the *P*-1 space group but two types of structures were found: [Pr(DMSO)_2_(OH_2_)_3_][Ru_2_(CO_3_)_4_(DMSO)(OH_2_)]·5H_2_O (**Prα**) (α structure) and [Ln(OH_2_)_5_][Ru_2_(CO_3_)_4_(DMSO)]·*x*H_2_O (Ln = Sm (**Smβ**), Gd (**Gdβ**)) (β structure). The α structure has a significantly lower density (2.142 g·cm^−^^3^) than the β structure (2.447, 2.426 g·cm^−^^3^, Table 1). The structure of [Sm(OH_2_)_4_][Ru_2_(CO_3_)_4_(OH_2_)]·2H_2_O (**Sm3D**) was determined by single crystal X-ray diffraction and is isostructural to the previously reported Gd, Eu and Yb derivatives (See Figures S3–S5) [21]. The PXRD of [Pr(OH_2_)_4_][Ru_2_(CO_3_)_4_(OH_2_)]·2H_2_O (**Pr3D**) shows that it is isostructural with Sm3D (See Figure S6).

**Figure 4:**
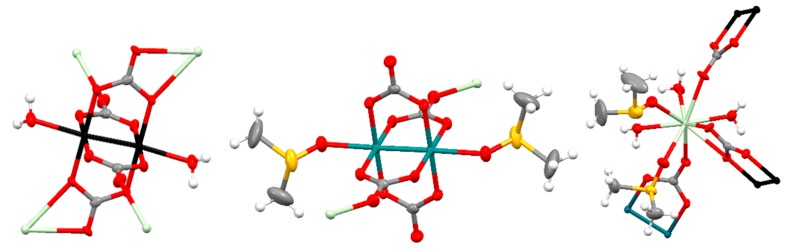
Representation (50% probability ellipsoids) of the coordination environments of the Ru_2_^5+^ (**left** and **center**) and Pr^3+^ (**right**) units that form the structure of **Prα**. Ruthenium: turquoise and black; praseodymium: pale green; oxygen: red; carbon: gray; sulfur: yellow; hydrogen: white. Ellipsoids of the hydrogen atoms are omitted for clarity.

The structure of **Prα** (α structure) is formed by [Ru_2_(CO_3_)_4_(OH_2_)_2_]^3−^, [Ru_2_(CO_3_)_4_(DMSO)_2_]^3−^ and [Pr(DMSO)_2_(OH_2_)_3_]^3+^ units in a 1:1:2 ratio giving a neutral 2D net (Figure 4). The two types of diruthenium units display a paddlewheel structure with two ruthenium atoms bridged by four carbonate ligands and two water or two DMSO molecules at the axial positions (Figure 4, left and center). Each carbonate ligand of the [Ru_2_(CO_3_)_4_(OH_2_)_2_]^3−^ units is also coordinated to a Pr^3+^ ions in such a way that two of the carbonates, in *trans* disposition, display a µ_3_-1κ*O*,2κ*O′*,3κ*O″* coordination mode while the other two carbonates display a µ_3_-1κ*O*,2:3κ^2^*O′O″*,*3*κ*O″* coordination mode (Figure 4, left). Two of the carbonate ligands, in *trans* disposition, of the [Ru_2_(CO_3_)_4_(DMSO)_2_]^3−^ bridge a Pr^3+^ ion and two ruthenium atoms with a µ_3_-1κ*O*,2κ*O′*,*3*κ*O″* coordination mode (Figure 4, center). The Ru–Ru distances are 2.264 and 2.258 Å, which are in the range 2.238–2.272 Å found for other tetracarbonatodiruthenium compounds [2,10,13,16,18,20,21]. The Pr^3+^ ions have a coordination number of nine and are surrounded by the three oxygen atoms of water molecules, two oxygen atoms of DMSO molecules and 4 oxygen atoms of 3 carbonate ligands (Figure 4, right).

**Figure 5:**
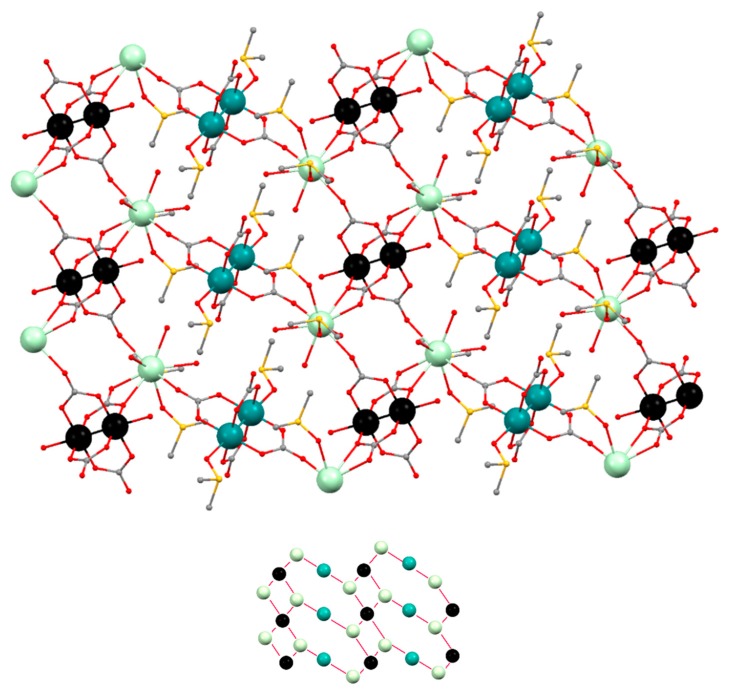
(**Top**): Ball and stick representation of the 2D structure of **Prα**. Ruthenium: turquoise and black; praseodymium: pale green; oxygen: red; carbon: gray; sulfur: yellow. Hydrogen atoms are omitted for clarity. (**Bottom**): Simplification of the 2D net. Turquoise: [Ru_2_(CO_3_)_4_(DMSO)_2_]^3−^ units; black: [Ru_2_(CO_3_)_4_(OH_2_)_2_]^3−^ units; pale green: [Pr(DMSO)_2_(OH_2_)_3_]^3+^ units.

The combination of the building blocks that form the structure of **Prα** gives rise to a 2D polymeric structure (Figure 5). If each type of Ru_2_^5+^ unit and the Ln^3+^ units are considered as nodes, the resulting net is built by triconected (Pr^3+^ ions, Figure 4 right), biconnected (Ru_2_^5+^ ions, Figure 4 center), and tetraconnected (Ru_2_^5+^ ions, Figure 4 left) nodes with a (2-c)(3-c)_2_(4-c) stoichiometry (Figure 5, bottom). Interestingly, the polymeric 2D structure of **Prα** is related to that of [Ln(OH_2_)_4_][Ru_2_(CO_3_)_4_(OH_2_)]·xH_2_O (Ln = Nd, Eu, Gd, Yb), **Pr3D** and **Sm3D [21]** that display a 3D net formed by mono and dimetallic nodes that are tri-, tetra- and hexaconnected (Figure S7).

Five water molecules per formula that do not belong to the 2D network have been found in the crystal structure of **Prα**. These water molecules establish multiple hydrogen bonds with neighbor water molecules and carbonate, DMSO and water ligands belonging to the 2D network. Interestingly, the thermogravimetric analysis of **Prα** (heating rate of 5 °C min^–1^, Figure S8) shows a weight loss in the 35–65 °C range that corresponds to ca. five water molecules per formula. Then, a plateau is observed until 90 °C, when a loss that corresponds to 3–4 water molecules is observed. The framework is stable until 190 °C when it begins to decompose. 

**Figure 6:**
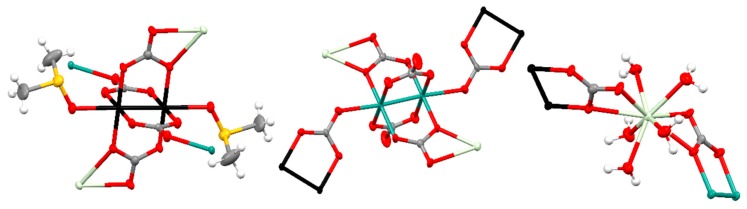
Representation (50% probability ellipsoids) of the coordination environments of the Ru_2_^5+^ (**left** and **center**) and Gd^3+^ (**right**) units that form the structure of **Gdβ**. Ruthenium: turquoise and black; gadolinium: pale green; oxygen: red; carbon: gray; sulfur: yellow; hydrogen: white. Ellipsoids of the hydrogen atoms are omitted for clarity.

The neutral layers that form the structure of **Smβ** and **Gdβ** are built with paddlewheel [Ru_2_(CO_3_)_4_(DMSO)_2_]^3−^ and [Ru_2_(CO_3_)_4_]^3−^ units and [Ln(OH_2_)_5_]^3+^ units combined in a 1:1:2 ratio. The ruthenium atoms in the two types of diruthenium fragments are bridged by four carbonate ligands and the axial positions are occupied by DMSO molecules or two carbonate ligands (Figure 6, left and center). Two *trans* carbonate ligands of the [Ru_2_(CO_3_)_4_(DMSO)_2_]^3−^ units are also coordinated to a Ln^3+^ ion displaying a µ_3_-1κ*O,*2:3κ*O′O″,3*κ*O″* coordination mode while the other *trans* carbonate ligands are coordinated to a Ru atom of the [Ru_2_(CO_3_)_4_]^3−^ units with a µ_3_-1κ*O*,2κ*O′*,*3*κ*O″* coordination mode (Figure 6, left). Two equatorial *trans* carbonate ligands of the [Ru_2_(CO_3_)_4_]^3−^ units are also coordinated to a Ln^3+^ ion with a µ_3_-1κ*O*,2:3κ*O′*,*3*κ*O″* coordination mode while the other two equatorial carbonate ligands do not bridge any additional metal ions (Figure 6, center). The Ru–Ru distances are in the 2.260–2.272 Å range, similar to other Ru–Ru distances reported for tetracarbonatodiruthenium compounds as mentioned above. The Ln^3+^ ions have a coordination number of nine with 5 oxygen atoms of 5 water molecules and 4 oxygen atoms of 2 carbonate ligands that belong to a [Ru_2_(CO_3_)_4_(DMSO)_2_]^3−^ and a [Ru_2_(CO_3_)_4_]^3−^ unit (Figure 6, right).

**Figure 7:**
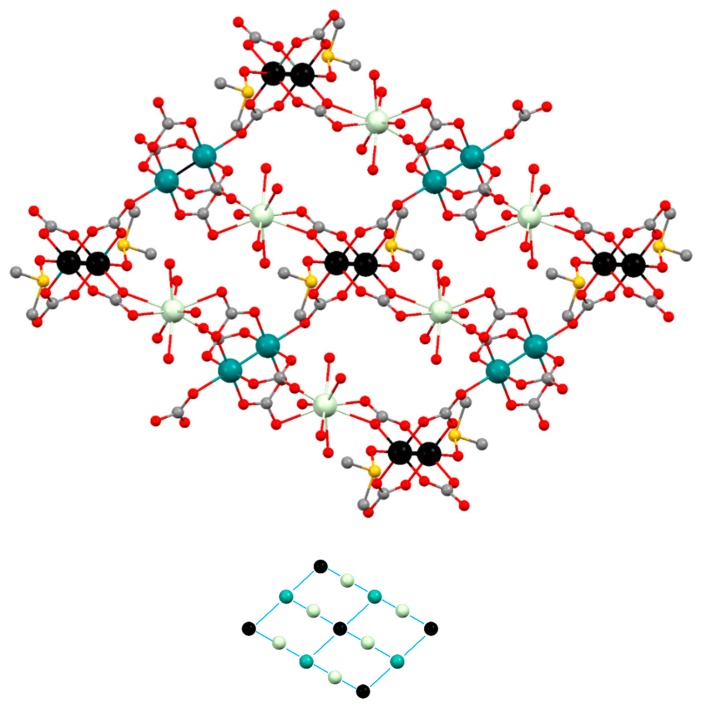
(**Top**): Ball and stick representation of the 2D structure of **Gdβ**. Ruthenium: turquoise and black; gadolinium: pale green; oxygen: red; carbon: gray; sulfur: yellow. Hydrogen atoms are omitted for clarity. (**Bottom**): Simplification of the 2D net. Turquoise: [Ru_2_(CO_3_)_4_]^3−^ units; black: [Ru_2_(CO_3_)_4_(DMSO)_2_]^3−^ units; pale green: [Gd(OH_2_)_5_]^3+·^ units.

**Smβ** and **Gdβ** display a grid-like structure, formed by biconnected nodes, [Ln(OH_2_)_5_]^3+^, and two types of tetraconnected nodes, [Ru_2_(CO_3_)_4_(DMSO)_2_]^3−^ and [Ru_2_(CO_3_)_4_]^3−^ units, with a (2-c)(3-c)_2_(4-c) stoichiometry (Figure 7).

Three crystallization water molecules per formula have been found in the structure of **Smβ**, while only two have been found in the structure of **Gdβ**. These water molecules form hydrogen bonds with neighbour water molecules and with carbonate and water ligands of the polymeric structure. The thermogravimetric analyses of **Smβ** and G**dβ** (heating rate of 5 °C min^–1^, Figures S9 and S10) show a gradual decomposition in the 35–200 °C range.

### 3.3. Magnetic Properties

**Figure 8:**
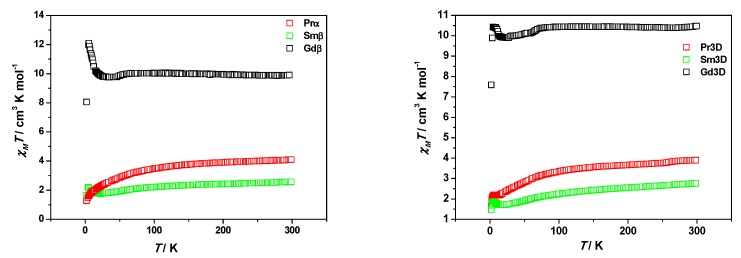
Plots of the *χ*_M_*T* vs. temperature for compounds **Prα**, **Smβ**, **Gdβ** (**left**) and **Pr3D**, **Sm3D** and [Gd(H_2_O)_4_][Ru_2_(CO_3_)_4_(H_2_O)_2_]·2.5H_2_O (**Gd3D**) [21] (**right**).

The temperature dependence of the magnetic susceptibility of **Prα**, **Smβ**, **Gdβ**, **Pr3D** and **Sm3D** was measured between 300 and 2 K at 1 T. The plots of the *χ*_M_*T* vs. temperature are displayed in Figure 8. Compounds with identical lanthanide present approximately the same *χ*_M_*T* values at room temperature despite their different crystal structure. Those values (4.10, 2.56, 10.40, 3.89 and 2.76 emu mol^−^^1^ K for **Prα**, **Smβ**, **Gdβ**, **Pr3D** and **Sm3D**, respectively) are slightly higher than the value expected from the sum of independent Ru_2_^5+^ and Ln^3+^ ions (3,48, 1.97 and 9.76 emu mol^−^^1^ K, respectively, for Pr^3+^, Sm^3+^ and Gd^3+^ with Ru_2_^5+^). 

The *χ*_M_*T* values for **Prα**, **Smβ**, **Pr3D** and **Sm3D** descend smoothly until ≈80 K. Below this temperature a sharper decrease is observed until 2 K for **Prα** and until 18, 12.6 and 12.7 K for **Smβ**, **Sm3D** and **Pr3D**, when the *χ*_M_*T* values increase and a maximum in the curves is observed at 5 K. However, there is almost no variation in the *χ*_M_*T* values for **Gdβ** until 60 K, even a slight increase can be detected. Then, the *χ*_M_*T* values decrease until 30 K and at lower temperatures they increase to reach a maximum of 12.10 emu mol^−^^1^ K at 5.4 K. Finally, *χ*_M_*T* values abruptly descend. This is the same pattern observed for [Gd(H_2_O)_4_][Ru_2_(CO_3_)_4_(H_2_O)_2_]·2.5H_2_O (**Gd3D**) [21] although the *χ*_M_*T* maximum is 10.43 emu mol^−^^1^ K at 4.6 K (Figure 8).

The decrease of *χ*_M_*T* has been observed in other heteronuclear tetracarbonatodiruthenium(II,III) derivatives in which the Ru_2_^5+^ centers are the sole magnetic species [7,9,13,14,19] and it has been ascribed to a large zero field splitting (ZFS) associated with the Ru_2_^5+^ species [7]. In **Prα**, **Smβ**, **Pr3D** and **Sm3D** this decrease is due to the sum of the ZFS of the Ru_2_^5+^ units and the depopulation of the *M*_J_ sublevels of the Ln(III) ions produced by the splitting of the ground state by the ligand field [22]. 

The **Gdβ** compound does not present an important temperature dependence of *χ*_M_*T* until low temperatures and, therefore, the contribution of Gd(III) to *χ*_M_*T* at high temperatures comes basically from the 7 unpaired electrons of the lanthanide ion that arise a ^8^S_7/2_ ground state, without first order spin-orbit coupling [23].

Intramolecular exchange coupling in lanthanide compounds is usually very weak due to the radially contracted nature of 4f orbitals [24]. Therefore, the increase in *χ*_M_*T* values at low temperatures could be ascribed to a canted ferrimagnetism produced by the diruthenium species. Actually, this phenomenon has been reported for several tetracarbonatodiruthenium compounds without other magnetic centers [7,9,13,14,19,21]. Interestingly, it was only observed for compounds in which two Ru_2_^5+^ species are connected by a carbonate ligand in the same fashion found in **Smβ**, **Gdβ**, **Pr3D** and **Sm3D**. However, a continuous lowering of *χ*_M_*T* values with temperature was observed when the axial position of the diruthenium species was occupied by other ligands. This is also the case for **Prα**.

The field dependence of the magnetization at 2 K between −5 and 5 T of compounds **Smβ** and **Gdβ** (Figures S11 and S12) shows almost saturation of the magnetization for **Gdβ** while the value of the magnetization is far from saturation at 5 T for **Smβ**. These measurements suggest the existence of predominant ferromagnetic interactions in **Gdβ** as in Mn_4_(H_2_O)_16_H[Ru_2_(CO_3_)_4_]_2_[Ru_2_(CO_3_)_4_(H_2_O)_2_]·11H_2_O and predominant canted ferrigmagnetism in **Smβ** as in K_x_H_1−x_[M(H_2_O)_4_][Ru_2_(CO_3_)_4_]·zH_2_O (M = Mg, Mn, Fe, Co, Ni) [18] and other tetracarbonatodiruthenium compounds without other magnetic centers [7,9,13,19,21]. In fact, Ru–O–Gd–O–Ru fragments with Ru–Gd distances of 4.352 and 4.445 Å are found in the structure of **Gdβ**.

The magnetic behavior of **Gdβ** has been fitted with Equation (1) [21], considering the sum of the contribution of the lanthanide ions following the free ion approximation, the contribution of diruthenium species taking into account a ZFS parameter (*D*) and a Weiss constant (*θ*) to consider intermolecular interactions. A TIP has also been added:

(1) $$\chi =\chi_{Ru}+\chi_{Ln}+TIP$$

where

$$\chi_{Ru}=\frac{Ng_{Ru}^{2}\mu_{B}^{2}}{3k(T-\theta )}\left[\frac{1+9e^{\frac{-2D}{kT}}}{4\left(1+e^{\frac{-2D}{kT}}\right)}+\frac{2+\frac{3kT}{2D}\left(1-e^{\frac{-2D}{kT}}\right)}{1+e^{\frac{-2D}{kT}}}\right]$$

and

$$\chi_{Ln}=\frac{Ng_{Ln}^{2}\mu_{B}^{2}}{3k(T-\theta )}J(J+1)$$

where *N*, *g µ*_B_ and *k* have the usual meanings.

The magnetic behavior of **Smβ** and **Sm3D** were fitted following a similar approach but considering the presence of excited states that can be thermally populated in the lanthanide ion. Thus, a spin-orbit parameter (*λ*) was considered for the Sm^3+^ ions as follows [23]:

$$\begin{array}{l} \chi_{Sm}=\frac{N\mu_{B}^{2}}{3kTx}[a_{1}x+b_{1}+\left(a_{2}x+b_{2}\right)e^{-7x/2}+\left(a_{3}x+b_{3}\right)e^{-8x}\\ +\left(a_{4}x+b_{4}\right)e^{-27x/2}+\left(a_{5}x+b_{5}\right)e^{-20x}\\ +\left(a_{6}x+b_{6}\right)e^{-55x/2}]/[3+4e^{-7x/2}\\ +5e^{-8x}+6e^{-27x/2}+7e^{-20x}+8e^{-55x/2}]\\ with\\ a_{1}=2.143\;\;b_{1}=7.347\\ a_{2}=42.92\;\;b_{2}=1.641\\ a_{3}=283.7\;\;b_{3}=-0.6571\\ a_{4}=620.6\;\;b_{4}=-1.9400\\ a_{5}=1122\;\;\;b_{5}=-2.835\\ a_{6}=1813\;\;b_{6}=-3.556 \end{array}$$

where *x* = *λ*/*kT*.

The equation to simulate the magnetic contribution of Pr^3+^ ions in **Prα** or **Pr3D** should consider the depopulation of the *M*_J_ sublevels which requires too many Hamiltonian Crystal Field parameters [22]. Therefore, we have used as an approximation the same model above employed for the Gd^3+^ derivatives.

The best data obtained from the fits are shown in Table 2 and the figures can be found in the SI (Figures S13–S18). The fits were made with the *χ*_M_*T* values from room temperature until the minimum of the *χ*_M_*T* vs. *T* curves. The *g*_Ru_ and *D* values obtained from the fits are within the normal range observed for diruthenium(II,III) compounds and are close to those for K_3_[Ru_2_(CO_3_)_4_]·4H_2_O, which were estimated to be 2.20 and 70 cm^−^^1^ [7]. However, the *D* value obtained for **Gdβ** was lower (39 cm^−^^1^) than expected. For this reason, a new fit was done with a fixed *D* value of 70 cm^−^^1^. In these cases, a higher *θ* and a lower *g* values were obtained.

## 4. Conclusions

The use of different solvents allows one to control the dimensionality of coordination polymers made from the reaction between K_3_[Ru_2_(CO_3_)_4_]·4H_2_O and Ln(NO_3_)_3_·xH_2_O (Ln^3+^ = Pr, Sm and Gd). The use of neat water leads to the formation of 3D coordination polymers while a H_2_O/DMSO mixture leads to the formation of 2D structures. A different 2D phase can be obtained depending of the reaction method. Thus, slow diffusion of the reagents gives a net made by triconnected Ln^3+^ nodes and two different Ru_2_^5+^ units that are bi- and tetraconnected when the Ln^3+^ ion is Pr^3+^ (α-structure, **Prα**). A grid-like net formed by biconnected Ln^3+^ nodes and two different tetraconnected Ru_2_^5+^ is obtained when the Ln^3+^ ions are Sm or Gd (β structure, **Smβ** and **Gdβ**). Direct mixing of the reagents leads to the α-structure in all cases.

The magnetic behavior of the complexes is consistent with the sum of the individual contributions of diruthenium and lanthanide species. The increase in *χ*_M_*T* at low temperatures is associated with a weak canted ferrimagnetism from the diruthenium species and weak ferromagnetic interaction between Ru_2_^5+^ and lanthanide ions.

## Figures and Tables

**Table 1:** Crystal and refinement data for **Prα**, **Smβ**, **Gdβ** and **Sm3D**.

Crystallographic Parameters	Prα	Smβ	Gdβ	Sm3D
Formula	PrRu_2_C_10_H_26_O_19_ S_3_·5H_2_O	SmRu_2_C_6_H_16_O_18_ S·3H_2_O	GdRu_2_C_6_H_16_O_18_ S·2H_2_O	SmRu_2_C_4_H_12_O_18_ 2H_2_O
fw	979.62	814.80	803.67	736.67
Space group	*P*-1	*P*-1	*P*-1	*C*2*/c*
*a*/Å	8.4508(4)	9.7951(5)	9.7687(6)	25.063(2)
*b*/Å	12.4403(6)	9.8502(5)	9.8237(6)	9.8420(8)
*c*/Å	15.5262(8)	12.8759(6)	12.8403(7)	14.0568(12)
α/°	78.243(1)	75.608(1)	75.657(1)	90
β/°	89.339(1)	70.296(1)	70.560(1)	95.092(2)
γ/°	72.147(1)	73.885(1)	73.951(1)	90
*V*/Å^3^	1518.81(13)	1107.0(10)	1100.07(11)	3453.7(5)
*Z*	2	2	2	8
*d* calc/*g*·cm^−3^	2.142	2.447	2.426	2.833
*μ*/mm^−1^	2.857	4.151	4.517	5.185
*R* indices (all data)	*R*_1_ = 0.0743 *wR*_2_ = 0.0974	*R*_1_ = 0.0504 *wR*_2_ = 0.1100	*R*_1_ = 0.0553 *wR*_2_ = 0.1241	*R*_1_ = 0.0585 *wR*_2_ = 0.0983
GooF on *F*^2^	1.082	1.039	1.073	1.052

**Table 2:** Magnetic parameters obtained for the fit of the magnetic data.

Compound	*g*_Ru_	*g*_Ln_^1^	*D* [cm^−1^]	*λ* [cm^−1^]	*θ* [K]	TIP [emu/mol]	σ ^2^
**Gdβ**	2.28	2.00	39		0.92	2.17 × 10^−4^	1.12 × 10^−2^
**Gdβ ^2^**	2.10	2.00	70		1.98	4.14 × 10^−12^	7.11 × 10^−3^
**Smβ**	2.18	0.29	73	256	1.73	5.27 × 10^−11^	5.12 × 10^−5^
**Sm3D**	2.17	0.29	77	270	1.11	7.76 × 10^−4^	4.88 × 10^−5^
**Prα**	2.07	0.80	78		−5.73	2.20 × 10^−3^	9.14 × 10^−3^
**Pr3D**	2.08	0.80	73		−7.09	3.27 × 10^−3^	7.95 × 10^−4^

^1^ These values were fixed in the fits. ^2^ Fixed *D* value.

## Supplementary Materials

The following are available online at https://www.mdpi.com/2073-4360/11/3/426/s1. Figure S1: IR spectrum of **Prα**. Figure S2: IR spectra of **Smβ** and **Gdβ**. Figure S3: View of the structure of **Sm3D** showing the different coordination environments. Figure S4: View along the *b* axis of a 1 × 1 × 1 packing of the structure of **Sm3D**. Figure S5: View along the *c* axis of a 1 × 1 × 1 packing of the structure of **Sm3D**. Figure S6: Experimental powder X-ray diffraction pattern obtained for **Pr3D** and calculated powder X-ray diffractogram simulated from the single crystal data of **Sm3D**. Figure S7: Simplification of the 2D net of **Prα** and simplification of the 3D net of [Ln(OH_2_)_4_][Ru_2_(CO_3_)_4_(OH_2_)]·xH_2_O (Ln = Nd, Eu, Gd, Yb), **Pr3D** and **Sm3D**. Figure S8: Thermogram of **Prα**. Figure S9: Thermogram of **Smβ**. Figure S10: Thermogram of **Gdβ**. Figure S11: Magnetization versus magnetic field between −5 T to 5 T for **Smβ**. Figure S12: Magnetization versus magnetic field between −5 T to 5 T for **Gdβ**. Figures S13–S18: Fits of the temperature dependence of the molar susceptibility *χ*_M_ and *χ*_M_T for all the compounds.
