# Supplementary material for: Tetracarbonatodiruthenium Fragments and Lanthanide(III) Ions as Building Blocks to Construct 2D Coordination Polymers
Source: Polymers (Basel). 2019 Mar 5;11(3):426. doi: 10.3390/polym11030426 (PMC6473724; doi:10.3390/polym11030426)
Supplement: Supplementary file 1 [file polymers-11-00426-s001.pdf]

Tetracarbonatodiruthenium Fragments and Lanthanide(III) Ions as Building Blocks to Construct 2D Coordination Polymers

Daniel Gutiérrez-Martín[1], Miguel Cortijo[1], Álvaro Martín-Humanes[1], Rodrigo González-Prieto[1], Patricia Delgado-Martínez[2], Santiago Herrero[1,*], José L. Priego[1,*], and Reyes Jiménez-Aparicio[1,*]

[1] Departamento de Química Inorgánica, Facultad de Ciencias Químicas, Universidad Complutense de Madrid, Ciudad Universitaria, E-28040 Madrid, Spain.

[2] Centro de Asistencia a la Investigación Difracción de Rayos X, Facultad de Ciencias Químicas, Universidad Complutense de Madrid, E-28040 Madrid, Spain.

* Correspondence: sherrero@ucm.es (S. H.); bermejo@ucm.es (J.L.P.); reyesja@ucm.es (R.J.-A.); Tel.: +34-913-94-5232 (S. H.); +34-913-94-4344 (J.L.P.); +34-913-94-4334 (R.J.-A.)

Received: date; Accepted: date; Published: date

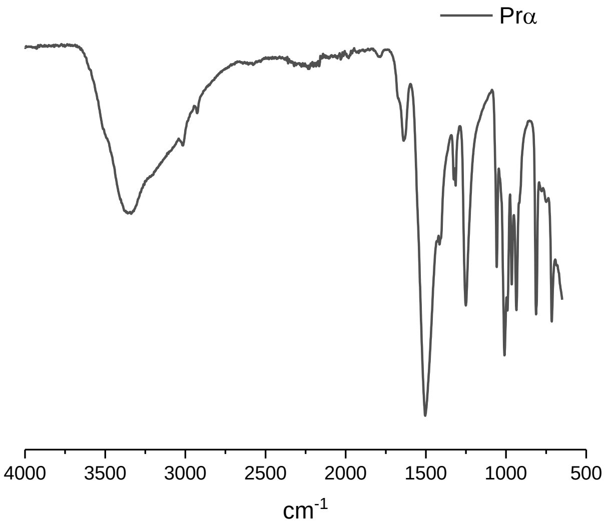

Figure S1. IR spectrum of **Prα**.

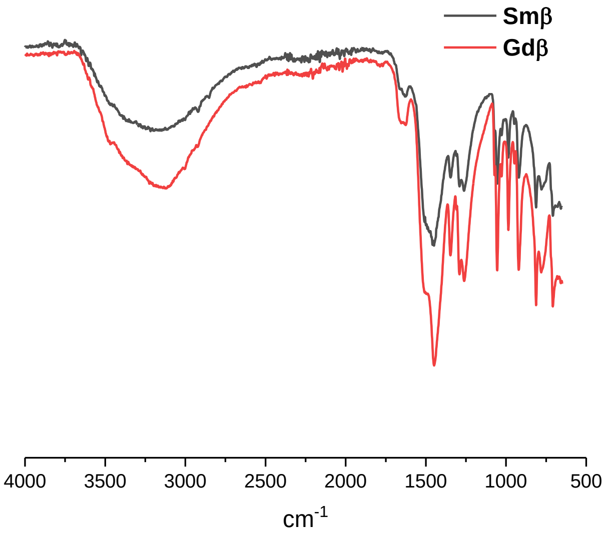

Figure S2. IR spectra of **Smβ** and **Gdβ**.

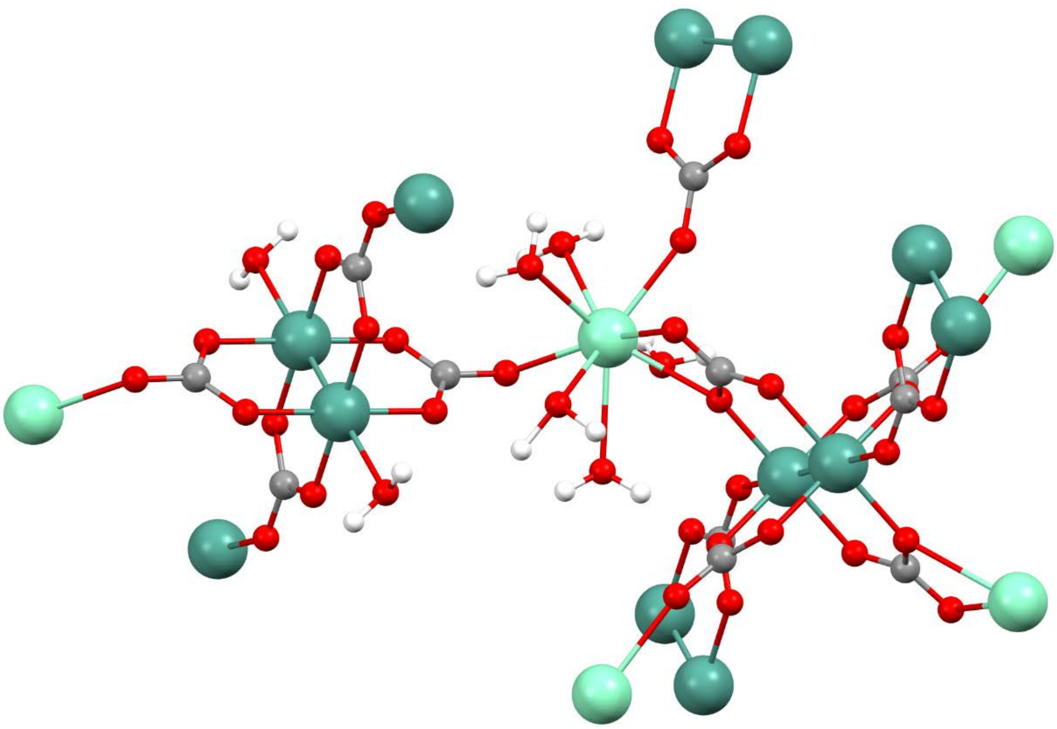

Figure S3. View of the structure of **Sm3D** showing the different coordination environments. Ruthenium: turquoise; samarium: pale green; oxygen: red; carbon: gray; hydrogen: white. Ellipsoids are omitted for clarity.

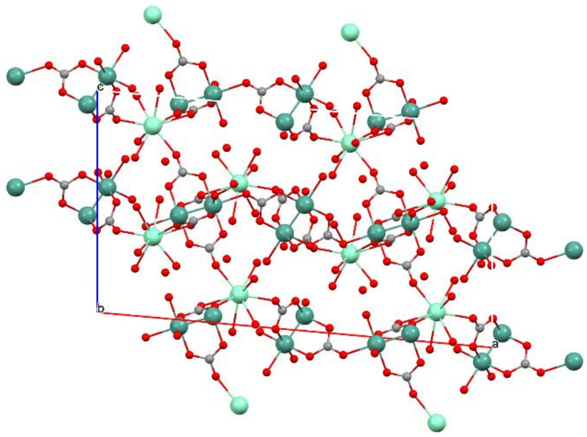

Figure S4. View along the *b* axis of a 1x1x1 packing of the structure of **Sm3D**.

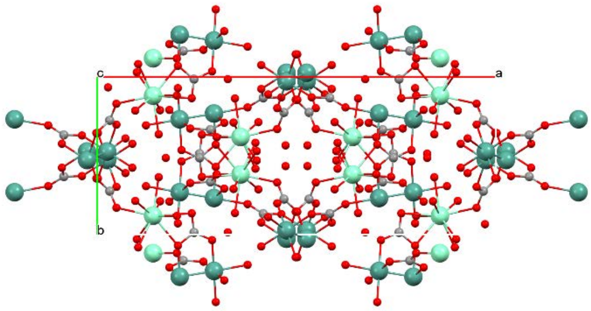

Figure S5. View along the *c* axis of a 1x1x1 packing of the structure of **Sm3D**.

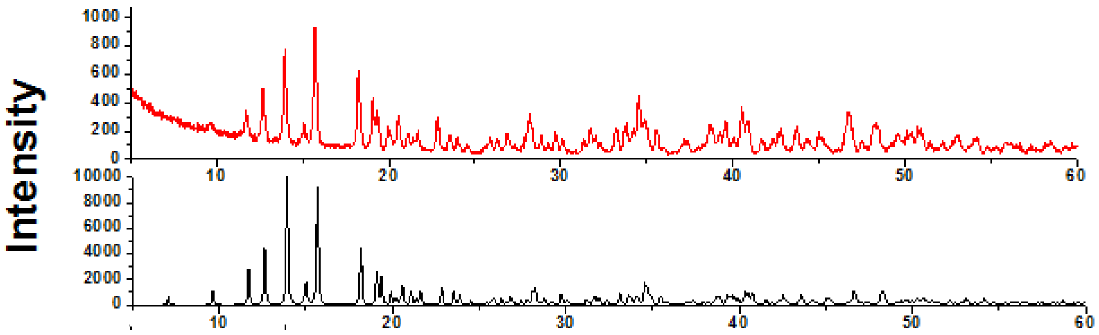

Figure S6. Experimental powder X-ray diffraction pattern obtained for **Pr3D** (red) and calculated powder X-ray diffractogram simulated from the single crystal data of **Sm3D** (black).

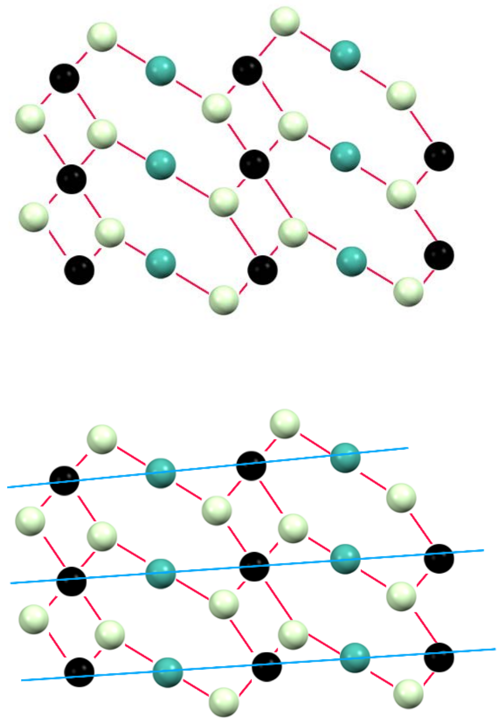

Figure S7. (Top): Simplification of the 2D net of **Prα**. (Bottom): Simplification of the 3D net of $[Ln(OH_2)_4][Ru_2(CO_3)_4(OH_2)]\cdot xH_2O$ (Ln = Nd, Eu, Gd, Yb), [1] **Pr3D** and **Sm3D**. Turquoise and black: Ru_2^{5+} units. Pale green: Ln^{3+}-units.

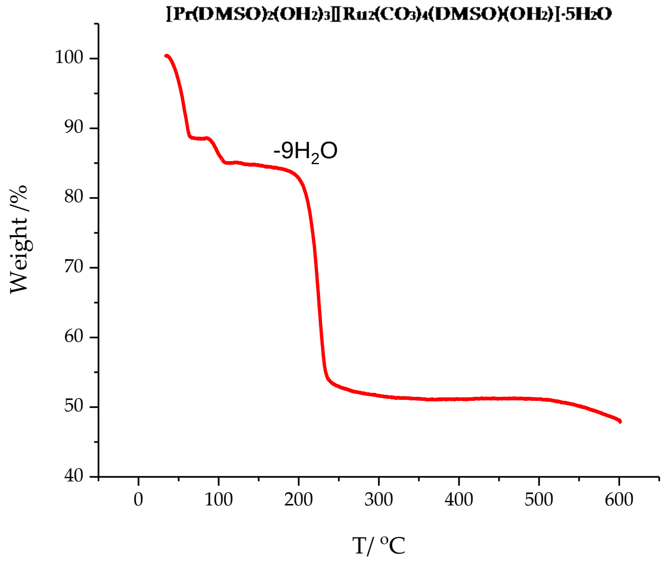

Figure S8: Thermogram of **Prα**.

[1] Delgado-Martínez, P.; González-Prieto, R.; Herrero, S.; Jiménez-Aparicio, R.; Perles, J.; Priego, J.L.; Torres, M.R.; Sufrate, B. Preparation of Crystalline Phases of 3D Coordination Polymers Based on Tetracarbonatodiruthenium Units and Lanthanide(III) Ions – Magnetic Characterization. *Eur. J. Inorg. Chem.* **2017**, 3161–3168. DOI:10.1002/ejic.201700281.

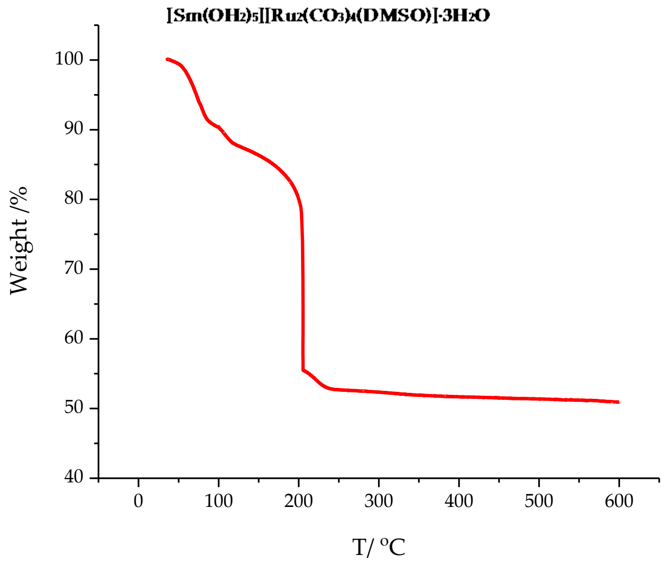

Figure S9: Thermogram of **Smβ**.

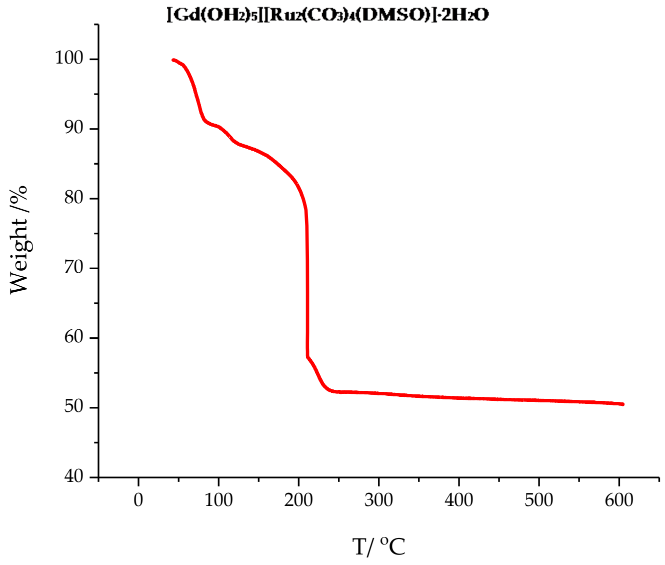

Figure S10: Thermogram of **Gdβ**.

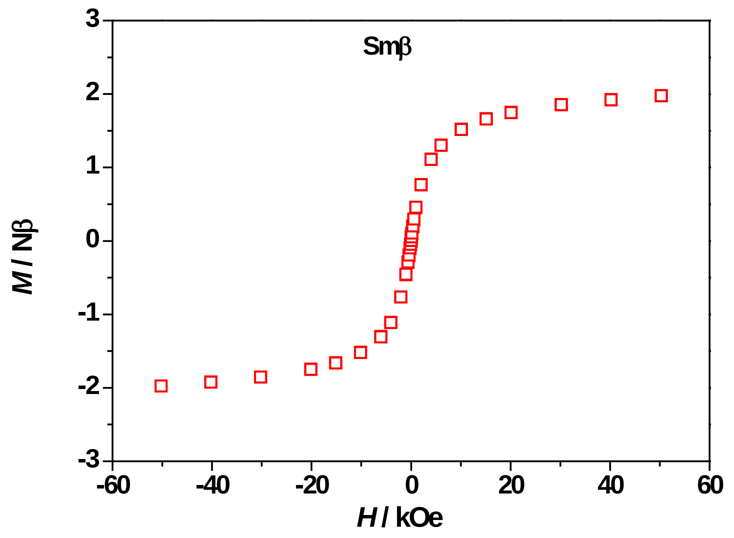

Figure S11. Magnetization versus magnetic field between -5 T to 5 T for **Smβ**.

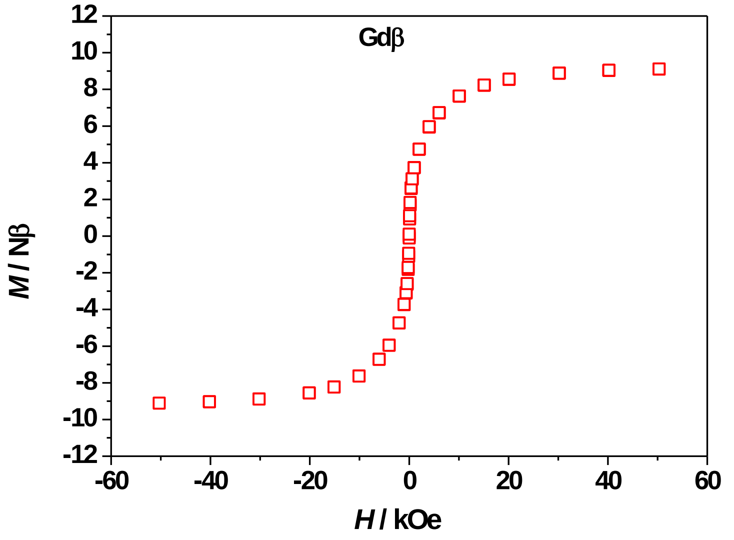

Figure S12. Magnetization versus magnetic field between -5 T to 5 T for **Gdβ**.

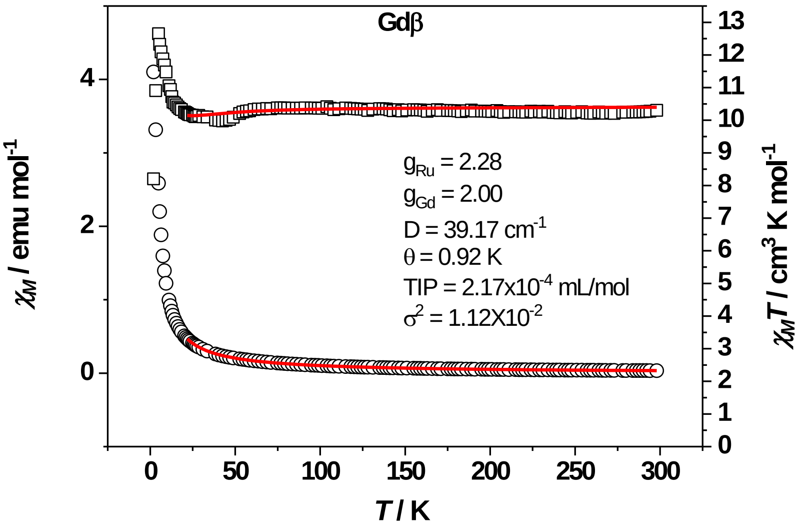

Figure S13: Temperature dependence of the molar susceptibility χ_M (circles) and $\chi_M T$ (squares) for **Gdβ**. Solid lines are the best fit to the model indicated in the text.

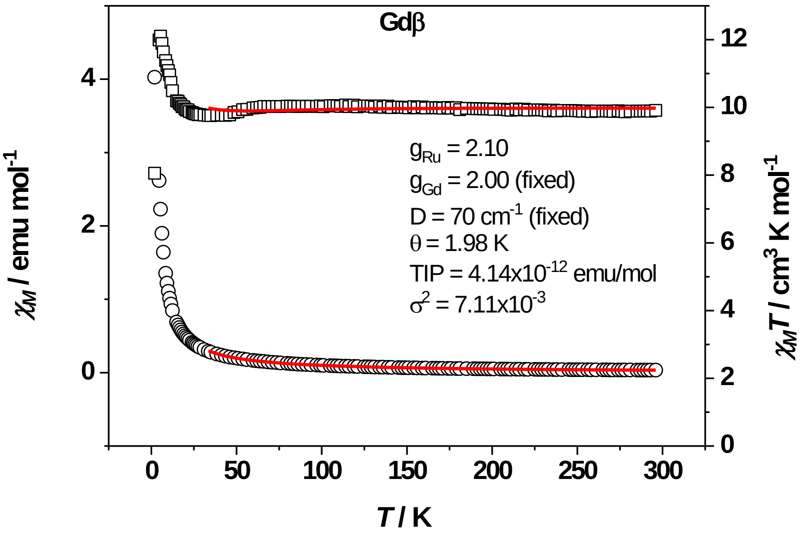

Figure S14. Temperature dependence of the molar susceptibility χ_M (circles) and $\chi_M T$ (squares) for **Gdβ**. Solid lines are the fit to the model indicated in the text with a fixed *D* value of 70 cm^{-1}.

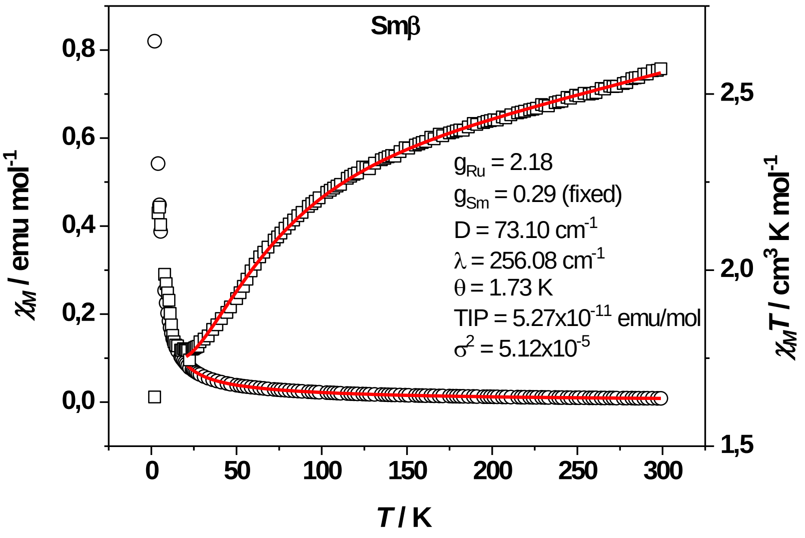

Figure S15: Temperature dependence of the molar susceptibility χ_M (circles) and $\chi_M T$ (squares) for **Smβ**. Solid lines are the best fit to the model indicated in the text.

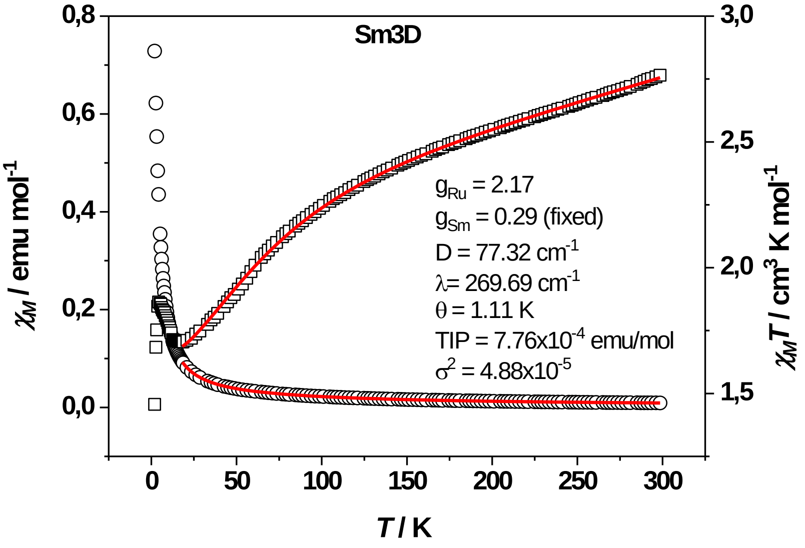

Figure S16: Temperature dependence of the molar susceptibility χ_M (circles) and $\chi_M T$ (squares) for **Pr3D**. Solid lines are the best fit to the model indicated in the text.

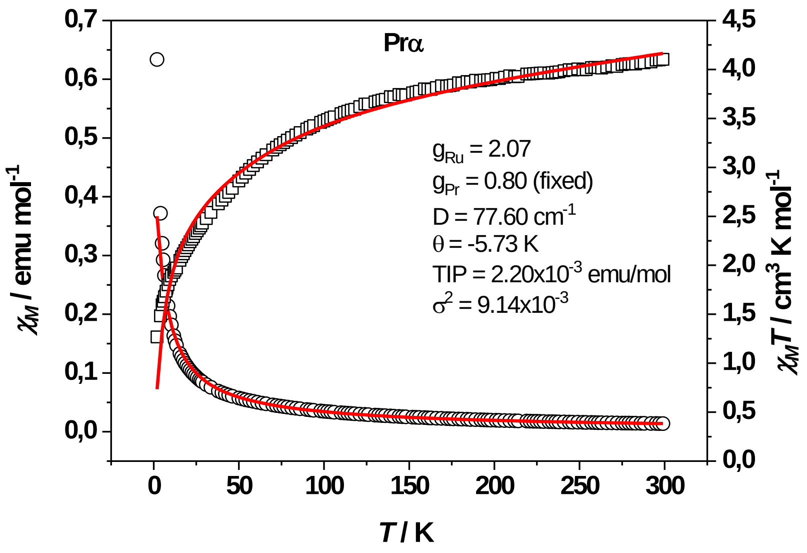

Figure S17: Temperature dependence of the molar susceptibility χ_M (circles) and χ_MT (squares) for **Prα**. Solid lines are the best fit to the model indicated in the text.

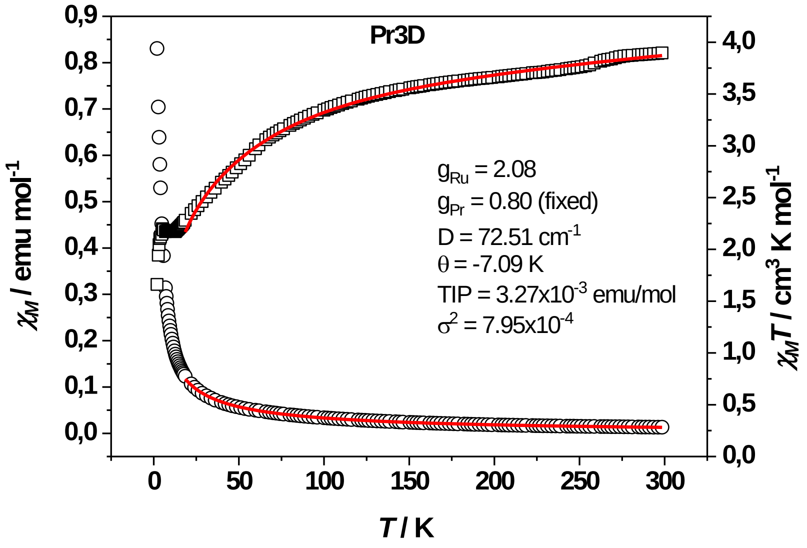

Figure S18: Temperature dependence of the molar susceptibility χ_M (circles) and χ_MT (squares) for **Pr3D**. Solid lines are the best fit to the model indicated in the text.
